# Correction: Inhibition of Spontaneous Recovery of Fear by mGluR5 after Prolonged Extinction Training

**DOI:** 10.1371/annotation/a71a5aba-ee14-439b-8952-69641642b71f

**Published:** 2013-06-27

**Authors:** Sheng-Chun Mao, Chih-Hua Chang, Chia-Chen Wu, Maria Juliana Orejanera, Olivier J. Manzoni, Po-Wu Gean

The name of the fourth author was spelled incorrectly. The correct name is: M. Juliana Orejarena. 

The correct citation is: Mao S-C, Chang C-H, Wu C-C, Orejarena MJ, Manzoni OJ, et al. (2013) Inhibition of Spontaneous Recovery of Fear by mGluR5 after Prolonged Extinction Training. PLoS ONE 8(3): e59580. doi:10.1371/journal.pone.0059580. 

